# Adolescents and young adults with sickle cell disease exhibit accelerated aging with elevated T-cell p16^INK4a^ expression

**DOI:** 10.18632/aging.206152

**Published:** 2024-11-14

**Authors:** Samuel R. Wilson, Natalia Mitin, Vanessa L. Ayer Miller, Andrew B. Smitherman, Marcus A. Carden

**Affiliations:** 1 Department of Medicine, Division of Hematology, University of North Carolina at Chapel Hill, Chapel Hill, NC 27514, USA; 2 Department of Pediatrics, Division of Pediatric Hematology/Oncology, University of North Carolina at Chapel Hill, Chapel Hill, NC 27514, USA; 3 UNC Blood Research Center, University of North Carolina at Chapel Hill, Chapel Hill, NC 27599, USA; 4 Sapere Bio, Research Triangle Park, Raleigh-Durham-Chapel Hill, NC 27709, USA; 5 Department of Pharmaceutical and Clinical Sciences, College of Pharmacy and Health Sciences, Campbell University, Buies Creek, NC 27506, USA; 6 Department of Epidemiology, Gillings School of Global Public Health, University of North Carolina at Chapel Hill, Chapel Hill, NC 27599, USA; 7 Cogent Biosciences, Waltham, MA 02451, USA

**Keywords:** sickle cell disease, aging, p16, adolescents, young adults

## Abstract

People living with sickle cell disease (SCD) experience complications indicative of an accelerated aging phenotype typified by early decline in physical function and increased risk for age-related conditions. Cellular senescence, measured by expression of p16^INK4a^ in peripheral T-lymphocytes, is recognized as one of the underlying contributors to organismal aging. To examine if cellular senescence is increased in SCD patients, we cross-sectionally measured and compared expression of p16 mRNA in peripheral blood T lymphocytes in 18 adolescents and young adults with SCD to 27 similarly aged individuals without SCD. Expression of p16 was dramatically higher in individuals with SCD vs. without SCD (10.1 vs. 8.7 log_2_ p16 units, respectively, *p* < 0.001) — a gap of 43 years in biological age — consistent with accelerated aging in the SCD population. Race was not associated with the increased p16 expression in the SCD group. These initial results suggest that individuals with SCD have a significantly higher cellular senescence burden which may contribute to premature aging, physiological decline, and excess morbidities. Additional longitudinal assessment and consideration for trials of senolytic therapies among individuals living with SCD and high p16 expression are warranted to improve their health span.

## INTRODUCTION

Life expectancy for individuals with sickle cell disease (SCD) is significantly lower than that of the general population; however, with advances in medical care, life expectancy has markedly improved over the past three decades in resource-rich countries [1]. Despite these gains, adolescents and adults with SCD still experience higher rates of aging-related morbidity and early mortality [2–4]. Individuals with SCD disproportionately experience functional decline, frailty, and increased rates of premature end-organ damage and malignancies [4–8]. While causes of these aging-related complications have not been elucidated, the presence of these complications suggests that many individuals with SCD experience accelerated aging, particularly in the bone marrow, possibly in response to chronic hypoxia, hemolysis and inflammation associated with a lifetime of ineffective erythropoiesis [3, 9, 10]. Earlier identification of individuals with SCD at greater risk for such age-related (and potentially life-threatening complications) is critical in improving long term outcomes and life expectancy in SCD.

Cellular senescence, a hallmark of biological aging, has been identified as a key contributor to functional decline and onset of aging-related diseases [11, 12]. Cells that undergo senescence are permanently cell-cycle arrested but remain metabolically active, secreting pro-inflammatory signals. Cellular senescence in the immune system has been shown to promote cellular senescence throughout organs and tissues and cause functional decline [13]. Expression of p16^INK4a^ (p16) is a reliable biomarker of cellular senescence [14]. In humans, increased expression of p16 mRNA in peripheral blood T lymphocytes (PBTL) is associated with well-established pro-aging exposures such as tobacco use, physical inactivity, chemotherapy, and radiation, and genetic alterations in the p16 locus have been associated with aging-related conditions in humans such as frailty, cardiovascular disease and type 2 diabetes [14, 15]. In adolescent and young adult survivors of cancer, PBTL p16 expression is markedly increased in a treatment dose-dependent manner and higher expression is associated with frailty and sarcopenia [16]. Taken together, prior work suggests that increased cellular senescence, as measured by PBTL p16 expression, identifies accelerated biological aging [12, 14, 17]. Given SCD is associated with an accelerated aging phenotype [6], we hypothesized that p16 expression would be elevated in people with SCD.

The primary objective of this study was to measure p16 expression in adolescents and young adults (AYAs) living with SCD and compare them to a pre-existing sample of similarly aged individuals without SCD. Our hypothesis was that individuals with SCD would have higher p16 expression compared to the non-SCD comparators.

## RESULTS

### Participant characteristics

Twenty-one AYAs with SCD were consented for this study, with three participants excluded from the analysis due to failed sample quality control for the p16 assay. Eighteen participants and 27 comparators were included in the final analysis. Participant and available comparator characteristics are summarized in Table 1, and additional SCD patient characteristics are detailed in Supplementary Table 1. Due to the need to protect the privacy of blood donors, there was no available demographic data beyond what is detailed in Table 1.

**Table 1:** Baseline characteristics of the study population.

	**SCD (*n* = 18)**	**Non-SCD Comparators (*n* = 27)**
Age (years), median (range)	22 (15–27)	20 (17–29)
Gender, *n* (%)		
Female	11 (61%)	20 (74%)
Male	7 (39%)	7 (26%)
Genotype, *n* (%)		
HbSS	16 (89%)	
HbSβ^0^-thalassemia	2 (11%)	
WBC count (10^9^/L), median (IQR)	11.3 (8.2–13.8)	
Hemoglobin (g/dL), median (IQR)	9.1 (8.7–9.6)	
Platelet count (10^9^/L), median (IQR)	356 (282–477)	
Absolute reticulocytes (10^9^/L), median (IQR)	223 (172–281)	
Absolute neutrophils (10^9^/L), median (IQR)	6.5 (4.6–8.2)	
Absolute lymphocytes (10^9^/L), median (IQR)	3.1 (1.9–3.5)	
On hydroxyurea, *n* (%)	10 (56%)	
Hydroxyurea dose^*^ (mg/kg)	19 (16–21)	
Time on hydroxyurea^*^ (months)	119 (64–140)	
On chronic transfusions, *n* (%)	8 (44%)	
Time on chronic transfusions^*^ (months), median (IQR)	59 (6–120)	

^*^Includes only participants receiving this therapy.

### Expression of p16

**Figure 1:**
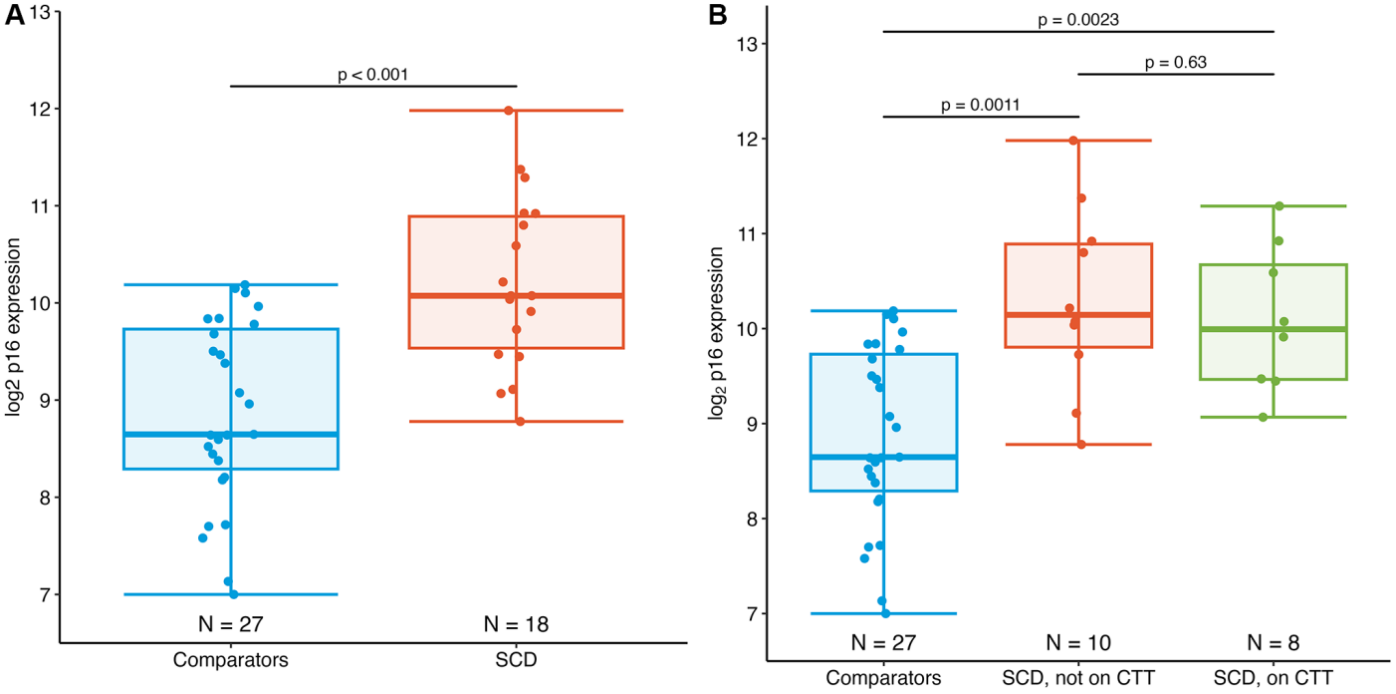
**Expression of PBTL p16^INK4a^ in AYAs with SCD.** (**A**) Mean p16 expression in AYAs with SCD (10.1 log_2_ p16 units) is significantly elevated compared to those without SCD (8.7 log_2_ p16 units), *p* < 0.001. (**B**) Expression of p16 in individuals with SCD did not differ by chronic transfusion therapy (CTT) status.

Participants with SCD had significantly higher p16 expression vs. comparators −10.1 vs. 8.7 log_2_ p16 units. Based on prior analysis of the regression of p16 expression by chronological age, this mean difference of 1.3 log_2_ units (95% CI 0.79–1.9, *p* < 0.001) between the SCD and comparator groups may be as much as 43 years (95% CI 23–63 years) of increased biological aging (Figure 1A) [17]. These results were similar when stratifying by chronic transfusion therapy (CTT), a therapy for severe SCD complications such as stroke. The mean difference in p16 expression between the SCD participants on CTT and comparators was 1.3 log_2_ p16 units (10.0 vs. 8.7 log_2_ p16 units, *p* = 0.002), while the difference between the SCD participants not on CTT and comparators was 1.4 (10.1 vs. 8.7 log_2_ p16 units, *p* = 0.001) (Figure 1B).

### Exploratory analysis

**Figure 2:**
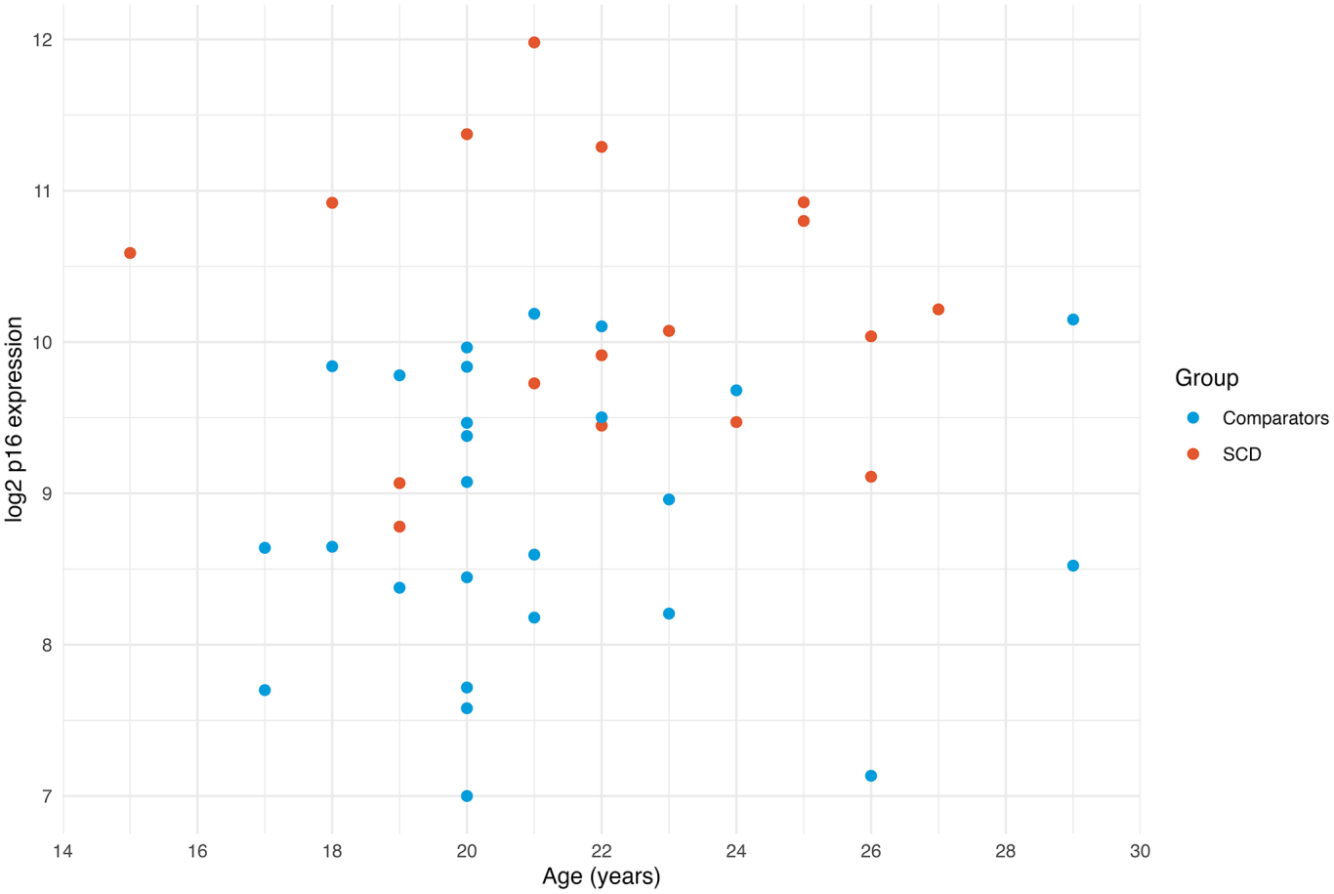
**Expression of p16 by age.** Scatterplot of p16 expression by age demonstrates that the SCD group generally has higher p16 expression across the entire age range. The youngest SCD participant has higher p16 expression than all the comparators.

Within the SCD group, we explored associations between laboratory measures, SCD complications, and PROs. In this small sample, there were no associations between any of these measures and p16 expression (Supplementary Table 2). In the SCD group, p16 expression was higher throughout, regardless of age (Figure 2).

## DISCUSSION

In this first assessment of PBTL p16 expression in SCD, we demonstrate that p16 expression, a marker of cellular senescence, is strikingly elevated in AYAs with SCD compared to non-SCD age matched comparators. The magnitude of this difference was unexpected, signifying an accelerated aging of up to 43 years in people with SCD, and is even larger than the difference between the AYA cancer survivors treated with pro-aging exposures (cytotoxic chemotherapy) and the same comparator group used in this study [16]. Our youngest participant, a 15-year-old with SCD, had a higher p16 expression than all the comparators, underscoring the early rise of p16 expression in this population. This work complements growing reports on accelerated aging in SCD [6, 18].

The etiology of the marked elevation in p16 expression in SCD is not yet known. Stress and ineffective erythropoiesis due to the hemolysis and increased RBC turnover, tissue hypoxia, anemia and chronic inflammation are all potential stressors to the bone marrow in people with SCD, which may manifest in accumulation of senescent cells and lead to accelerated organismal aging [9, 19].

To protect the privacy of the blood donors, the core of the comparator group, self-identified race of these participants is not available. We explored whether the marked difference in p16 expression observed in this study between the two groups could be partially explained by racial differences, as >90% of individuals with SCD identify as Black/African-American. There is no convincing evidence that p16 expression varies by race. Prior studies have observed no difference in p16 expression between Black/African-American and Caucasian populations [15]. To further explore, we compared p16 expression in the SCD cohort to nine similarly-aged individuals who self-identified as Black/African-American from the previous AYA cancer survivor p16 study, all of whom had received prior chemotherapy [20]. This comparison showed higher mean p16 expression in the SCD cohort (10.1 vs. 8.8 log_2_ units, *p* = 0.2), albeit the difference was not statistically significant likely due to the small sample (see Supplementary Figure 1). Subgroup comparisons within the AYA cancer survivor cohort also showed no differences by race. We also observed no overall differences in p16 expression by race after combining participants from multiple datasets from prior p16 studies (see Supplementary Figure 2). Altogether, these findings support that race alone does not account for the surprisingly large differences in p16 expression observed in this study.

Lastly, we explored any association between p16 expression and clinical/PRO measures in SCD participants in this small SCD patient sample to yield insight into potential drivers and clinical significance. As shown in Supplementary Table 2, we did not detect an association between p16 expression and any of the assessed clinical and PRO measures. Additionally, there was no association between disease-modifying therapies such as hydroxyurea, chronic transfusion therapy and p16 expression. There are several potential explanations for these findings: (1) This study was underpowered to detect these associations, (2) the p16 expression observed is a predictive marker of future morbidity and thereby may be more suitable for a longitudinal cohort, or most likely (3) the widespread impact and burden of SCD on cellular senescence itself may be greater than these various markers of clinical severity, which often does not capture disease severity in SCD, which is poorly defined [21]. We anticipate that a minimum sample size of at least 80–90 SCD participants is necessary to have 80% power to detect these associations.

These findings must be interpreted in the setting of study limitations. A cross-sectional design and limited sample size may not capture the relationship between p16 expression and treatments and clinical outcomes. A larger longitudinal study may be able to dissect causes of elevated p16 expression and may lead to interventional studies to address accelerated aging in SCD. Additionally, the bone marrow compartment in people living with SCD may be more prone to the accumulation of senescent cells, making PBTL p16 expression an incomplete reflection of organismal aging in this population. Thus, the age gap we found in this study may be an overestimate. Future studies should also examine the correlation of PBTL p16 expression with other senescence and immune markers from other body compartments in individuals with SCD.

## CONCLUSIONS

We demonstrate that AYAs with SCD exhibit early and accelerated biological aging with markedly increased p16 expression, a marker of cellular senescence. While further prospective research is needed to establish the clinical implications and predictive utility for elevated p16 expression in patients with SCD, these initial findings provide molecular evidence of biological aging beginning at a young age in individuals living with SCD.

## METHODS

### Study design and participants

The study cross-sectionally measured p16 expression in both individuals with and without SCD. The study protocol was approved by the Institutional Review Board at the University of North Carolina (UNC). AYAs with SCD (HbSS or HbSβ^0^-thalassemia genotypes only) ages 15 to 29 years receiving care at UNC SCD clinics from 2021–2022 were enrolled following informed consent and assent as appropriate. A 10mL peripheral blood specimen was collected for p16 analysis and participants completed patient-reported outcome (PRO) measures validated for use among people with SCD. We used a convenient sample of AYAs without SCD comparators that was previously recruited from 2018–2020 for an AYA cancer survivor study of p16 expression [16]. These comparators were either recruited from the UNC Platelet Donation Center (healthy individuals) or from the UNC Children’s Hematology Clinic (seen for non-malignant, non-SCD disorders such as iron deficiency anemia) and provided a blood sample similarly to individuals with SCD.

### Measures

For all participants, p16 expression was measured as previously described [14–16, 22]. Briefly, CD3+ cells were purified from whole blood and used to isolate total RNA. Expression of p16 was measured by a TaqMan RT-PCR (Sapere Bio, Research Triangle Park, NC, USA) and reported as log_2_ units. Collection of key patient and treatment data was performed by medical record abstraction. In non-SCD samples where p16 expression was below the limit of detection, the value of 7 log_2_ p16 units (lower limit of assay detection) was assigned. None of the SCD samples had p16 expression below the limit of detection.

### Statistical analysis

Expression of p16 in log_2_ units was compared among groups using 2-sided Student’s *t*-tests. Other measures were compared to p16 expression with linear regression. Analysis was performed with R (R Foundation for Statistical Computing, Vienna, Austria). Statistical significance was defined as *p*-value < 0.05 with 95% confidence intervals (CI) reported for all tests. See Supplementary Methods for additional details.

### Data sharing statement

De-identified data from this study will be shared upon reasonable request via email to the corresponding author.

## Supplementary Materials

Supplementary Methods

Supplementary Figures

Supplementary Tables
