# Supplementary material for: Adolescents and young adults with sickle cell disease exhibit accelerated aging with elevated T-cell p16INK4a expression
Source: Aging (Albany NY). 2024 Nov 14;16(21):13225–36. doi: 10.18632/aging.206152 (PMC11719104; doi:10.18632/aging.206152)
Supplement: Supplementary Methods [file aging-16-206152-s001.pdf]

SUPPLEMENTARY METHODS

Surveys

Following informed consent, participants aged 18 and over completed the (1) Adult Sickle Cell Quality of Life Measurement Information System (ASCQ-Me) surveys, including the Sickle Cell Medical History Checklist, Emotional Impact Computer Adaptive Test (CAT) v2.0, Pain Impact CAT v2.0, Social Functioning Impact CAT v2.0, Sleep Impact CAT v2.0, Stiffness Impact CAT v2.0, and Pain Episodes Frequency and Severity and (2) Patient-Reported Outcomes Measurement Information System® (PROMIS) 29 Profile v2.0. For participants under age 18, participants or guardians completed the PROMIS® Pediatric Profile v2.0 Profile-25, depending on patient or guardian preference. A peripheral blood specimen was then collected for p16 expression testing. Comparators were recruited from the UNC Platelet Donation Center and the UNC Children's Hematology Clinic (seen for non-malignant, non-SCD disorders such as iron deficiency anemia).

Measurement of p16 expression

p16 expression analysis was performed by Sapere Bio (Research Triangle Park, NC) using a previously described protocol [1–4]. Each run included positive and negative controls to monitor assay performance. Expression of p16 among age-matched comparators without SCD was collected in a prior study, as previously described [1]. As p16 expression is generally undetectable in healthy individuals under age 20 [5], comparator samples that had undetectable p16 expression were assigned the lower limit of detection of 7 $\log_2$ units for data visualization.

Other measures

SCD-related comorbidities, hydroxyurea dose, chronic transfusion therapy use, and laboratory measures were obtained through chart review from the electronic medical record. Laboratory measures such as white blood cell (WBC) count, hemoglobin, platelet count, absolute reticulocyte count (ARC), and absolute neutrophil count (ANC) were extracted from the most recent visit, usually the day of enrollment. SCD genotype was confirmed via chart review. Each of the ASCQ-Me and PROMIS surveys were scored per the corresponding manual, and the total score for each survey was used as a continuous measure. The standardized score for each of these surveys has a mean of 50 and a standard deviation of 10. For the ASCQ-Me measures, higher scores suggest healthier or improved function, except for the Pain Episode Frequency and Severity measures in which higher scores signify increased pain burden [6]. For the PROMIS-29 profile, higher scores for Pain Interference, Depression, Fatigue, Anxiety, and Sleep Disturbance signify worse health, while higher scores for Physical Function and Ability to Participate in Social Roles and Activities correspond to improved function [7].

SUPPLEMENTARY REFERENCES

1. Smitherman AB, Wood WA, Mitin N, Ayer Miller VL, Deal AM, Davis IJ, Blatt J, Gold SH, Muss HB. Accelerated aging among childhood, adolescent, and young adult cancer survivors is evidenced by increased expression of $p16^{INK4a}$ and frailty. Cancer. 2020; 126:4975–83. https://doi.org/10.1002/cncr.33112 PMID:32830315

2. Liu Y, Sanoff HK, Cho H, Burd CE, Torrice C, Ibrahim JG, Thomas NE, Sharpless NE. Expression of p16(INK4a) in peripheral blood T-cells is a biomarker of human aging. Aging Cell. 2009; 8:439–48. https://doi.org/10.1111/j.1474-9726.2009.00489.x PMID:19485966

3. Sanoff HK, Deal AM, Krishnamurthy J, Torrice C, Dillon P, Sorrentino J, Ibrahim JG, Jolly TA, Williams G, Carey LA, Drobish A, Gordon BB, Alston S, et al. Effect of cytotoxic chemotherapy on markers of molecular age in patients with breast cancer. J Natl Cancer Inst. 2014; 106:dju057. https://doi.org/10.1093/jnci/dju057 PMID:24681605

4. Wood WA, Krishnamurthy J, Mitin N, Torrice C, Parker JS, Snavely AC, Shea TC, Serody JS, Sharpless NE. Chemotherapy and Stem Cell Transplantation Increase $p16^{INK4a}$ Expression, a Biomarker of T-cell Aging. EBioMedicine. 2016; 11:227–38. https://doi.org/10.1016/j.ebiom.2016.08.029 PMID:27591832

5. Muss HB, Smitherman A, Wood WA, Nyrop K, Tuchman S, Randhawa PK, Entwistle AR, Mitin N, Shachar SS. p16 a biomarker of aging and tolerance for cancer therapy. Transl Cancer Res. 2020; 9:5732–42. https://doi.org/10.21037/tcr.2020.03.39 PMID:35117935

6. Keller S, Yang M, Evensen C, Cowans T. ASCQ-ME User's Manual. Washington, DC: American Institutes for Research. 2017.

7. PROMIS Adult Profile Instruments Scoring Manual. 2021. https://www.healthmeasures.net/images/PROMIS/manuals/Scoring_Manuals_/PROMIS_Adult_Profile_Scoring_Manual.pdf.
