# Supplementary material for: Adolescents and young adults with sickle cell disease exhibit accelerated aging with elevated T-cell p16INK4a expression
Source: Aging (Albany NY). 2024 Nov 14;16(21):13225–36. doi: 10.18632/aging.206152 (PMC11719104; doi:10.18632/aging.206152)
Supplement: Supplementary Figures [file aging-16-206152-s002.pdf]

SUPPLEMENTARY FIGURES

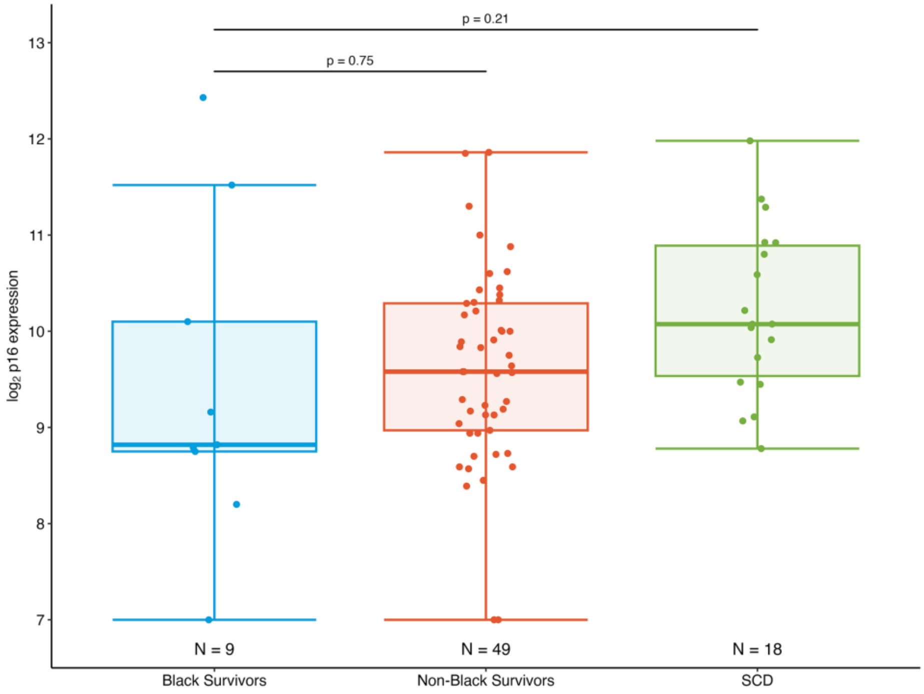

Supplementary Figure 1. Comparison of mean p16 expression in self-identified Black/African-American cancer survivors, non-Black survivors, and the sickle cell disease (SCD) groups show that individuals with SCD have a distinctively higher mean p16 expression (not significant). These results imply that race alone does not account for the higher mean p16 expression in SCD observed in this study.

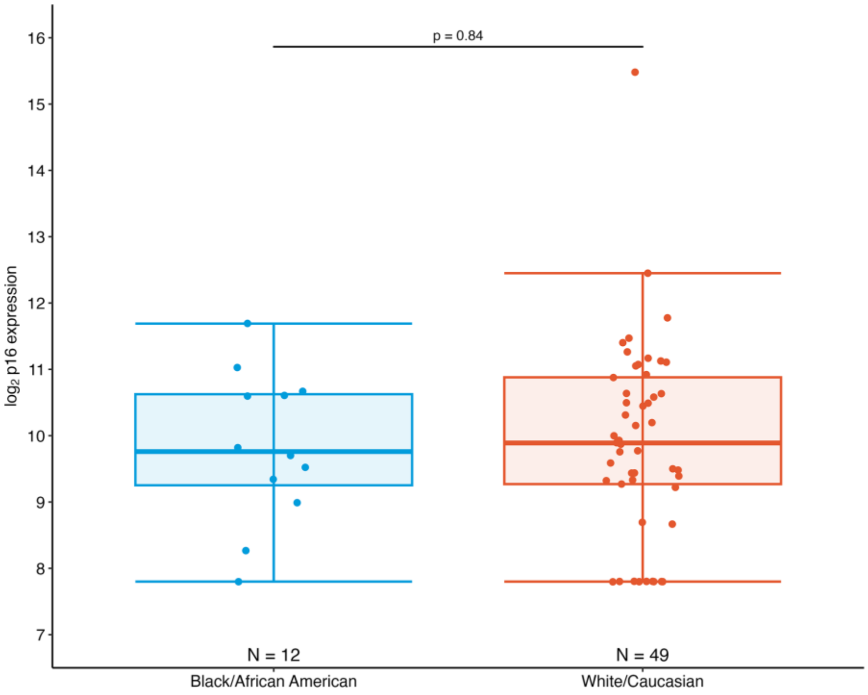

Supplementary Figure 2. Cross-sectional compilation of similarly aged cohort either with cancer prior to therapy or healthy comparators demonstrate no difference in p16 expression between self-identified Black/African-American and White/Caucasians.
