# Supplementary material for: Adolescents and young adults with sickle cell disease exhibit accelerated aging with elevated T-cell p16INK4a expression
Source: Aging (Albany NY). 2024 Nov 14;16(21):13225–36. doi: 10.18632/aging.206152 (PMC11719104; doi:10.18632/aging.206152)
Supplement: Supplementary Tables [file aging-16-206152-s003.pdf]

SUPPLEMENTARY TABLES

Supplementary Table 1. Extended SCD participant characteristics.

Characteristic	Overall, *N* = 18	Not on CTT (*N* = 10)	On CTT (*N* = 8)
Age (years), median (range)	22 (15–27)	22 (18–27)	22 (15–25)
Gender, *n* (%)			
Female	11 (61%)	6 (60%)	5 (62%)
Male	7 (39%)	4 (40%)	3 (38%)
Genotype, *n* (%)			
HbSS	16 (89%)	9 (90%)	7 (88%)
HbSβ^0-thalassemia	2 (11%)	1 (10%)	1 (12%)
WBC count (10^9/L), median (IQR)	11.3 (8.2–13.8)	10.9 (8.1–13.3)	11.3 (10.2–13.8)
Hemoglobin (g/dL), median (IQR)	9.1 (8.7–9.6)	9.1 (8.5–9.2)	9.4 (8.9–10.0)
Platelet count (10^9/L), median (IQR)	356 (282–477)	306 (181–350)	472 (435–554)
Absolute reticulocytes (10^9/L), median (IQR)	223 (172–281)	199 (164–244)	259 (175–290)
Absolute neutrophils (10^9/L), median (IQR)	6.5 (4.6–8.2)	5.3 (3.6–7.1)	8.1 (6.2–8.2)
Absolute lymphocytes (10^9/L), median (IQR)	3.05 (1.9–3.5)	3.25 (2.8–3.6)	2.2 (1.8–3.4)
On hydroxyurea, *n* (%)	10 (56%)	8 (80%)	2 (25%)
Hydroxyurea dose* (mg/kg), median (IQR)	19 (16–21)	19 (18–22)	16 (14–18)
Time on hydroxyurea* (months), median, (IQR)	119 (64–140)	119 (50–135)	126 (105–147)
Time on CTT (months)*, median (IQR)	59 (6–120)		59 (6–120)
History of acute chest syndrome, *n* (%)	15 (83%)	8 (80%)	7 (88%)
History of stroke, *n* (%)	4 (22%)	0 (0%)	4 (50%)
History of priapism, *n* (% of males)	4 (67%)	3 (75%)	1 (50%)
History of splenic sequestration, n (%)	9 (53%)	5 (56%)	4 (50%)
ASCQ-Me Measures, median (IQR)			
Emotional Impact	57 (50–64)	58 (56–66)	54 (49–59)
Pain Impact	59 (44–67)	67 (46–70)	48 (44–61)
Social Functioning Impact	61 (50–69)	63 (51–69)	61 (47–65)
Sleep Impact	55 (50–58)	55 (50–64)	56 (42–58)
Stiffness Impact	63 (49–66)	66 (64–70)	54 (47–59)
Pain Episode Frequency	52 (40–56)	54 (45–56)	44 (34–58)
Pain Episode Severity	45 (43–52)	45 (43–53)	50 (44–52)
PROMIS-29 Profile, median (IQR)			
Pain Interference	42 (42–61)	42 (42–56)	52 (42–63)
Depression/Sadness	49 (41–55)	41 (41–52)	51 (50–56)
Physical Function	48 (40–57)	57 (46–57)	43 (40–52)
Ability to Participate in Social Roles and Activities	52 (52–64)	55 (52–64)	52 (47–64)
Fatigue	49 (42–57)	45 (34–56)	49 (47–60)
Anxiety/Fear	51 (40–56)	40 (40–56)	52 (50–56)
Sleep Disturbance	53 (46–58)	52 (46–54)	53 (46–63)

*Includes only participants receiving this therapy. ASCQ-Me: Adult Sickle Cell Quality of Life Measurement Information System; PROMIS: Patient-Reported Outcomes Measurement Information System; CTT: chronic transfusion therapy.

Supplementary Table 2. Association between p16 and participant characteristics in the SCD group.

Characteristic	*N*	Beta	95% CI	*p*-value
Age	18	−0.03	−0.17–0.12	0.7
WBC count	18	0.07	−0.04–0.18	0.2
Hemoglobin	18	0.07	−0.54–0.68	0.8
Platelet count	18	0.00	0.00–0.00	0.5
Absolute Reticulocytes	17	0.00	0.00–0.01	0.6
Absolute Neutrophil count	18	0.11	−0.04–0.25	0.14
Absolute Lymphocyte count	18	0.02	−0.35–0.39	>0.9
Total Bilirubin	18	0.12	−0.07–0.30	0.2
Creatinine	18	−0.24	−1.3–0.82	0.6
On Hydroxyurea	18	0.25	−0.66–1.2	0.6
Hydroxyurea dose	10	−0.02	−0.12–0.08	0.7
Time on hydroxyurea therapy (months)	10	0.00	−0.02–0.01	0.6
History of acute chest syndrome	18	0.61	−0.56–1.8	0.3
History of stroke	18	−0.48	−1.5–0.58	0.4
ASCQ-Me Emotional Impact	17	−0.01	−0.05–0.04	0.8
ASCQ-Me Pain Impact	17	0.00	−0.03–0.04	0.8
ASCQ-Me Social Functioning Impact	17	−0.02	−0.05–0.02	0.4
ASCQ-Me Sleep Impact	17	0.01	−0.03–0.05	0.6
ASCQ-Me Stiffness Impact	17	0.00	−0.05–0.05	>0.9
ASCQ-Me Pain Episode Frequency	17	0.03	−0.02–0.07	0.2
ASCQ-Me Pain Episode Severity	17	0.00	−0.05–0.05	>0.9
PROMIS-29 Pain Interference	17	0.00	−0.04–0.05	0.9
PROMIS-29 Depression/Sadness	17	0.02	−0.03–0.07	0.3
PROMIS-29 Physical Function	17	−0.02	−0.08–0.04	0.4
PROMIS-29 Ability to Participate in Social Roles/Activities	17	−0.02	−0.08–0.03	0.4
PROMIS-29 Fatigue	17	0.00	−0.03–0.04	0.8
PROMIS-29 Anxiety	17	−0.01	−0.07–0.04	0.5
PROMIS-29 Sleep Disturbance	17	0.00	−0.04–0.04	0.9
